# c-MET在非小细胞肺癌中的作用机制及其治疗和检测

**DOI:** 10.3779/j.issn.1009-3419.2015.12.06

**Published:** 2015-12-20

**Authors:** 红格 梁, 孟昭 王

**Affiliations:** 100730 北京，中国医学科学院中国协和医科大学北京协和医学院呼吸内科 Chinese Academy of Medical Sciences, Chinese Peking Union Medical College, Peking Union Medical College Hospital, Beijing 100730, China

**Keywords:** 肺肿瘤, c-MET, HGF, 治疗, 检测, Lung neoplasms, c-MET, HGF, Treatment, Testing

## Abstract

肝细胞生长因子/c-MET（hepatocyte growth factor/c-MET, HGF/c-MET）信号通路可通过多种机制如*c-MET*突变、扩增、过表达和HGF过表达被异常激活，并在非小细胞肺癌（non-small cell lung cancer, NSCLC）的发生发展以及表皮生长因子受体抑制剂（epidermal growth factor receptor tyrosine kinase inhibitor, EGFR-TKI）的耐药性方面发挥重要作用。因此，c-MET是继EGFR、ALK之后NSCLC的又一分子治疗靶点。目前c-MET抑制剂在一些临床试验中表现出了良好的的应用前景，但其在临床应用中的有效性和安全性的评估，以及对于c-MET检测方法的选择及判定标准还需进一步讨论。本文就c-MET在NSCLC中的作用机制、治疗前景和检测方法的选择作一综述。

## Abstract (en)

Hepatocyte growth factor/c-MET (HGF/c-MET) signaling pathway can be abnormal activated by many mechanisms such as *c-MET* mutation, amplification and the overexpression of HGF, and it plays an important role in the development of non-small cell lung cancer (NSCLC), as well as in the tolerance of epidermal growth factor receptor tyrosine kinase inhibitor (EGFR-TKI) in NSCLC. Therefore, c-MET is a new molecular target for the therapy of NSCLC since EGFR and ALK. At present, although the c-MET inhibitors have shown a potential prospect in some clinical trials, its assessment of safety and effectiveness in clinical applications, and the choice of testing methods and standards still need a further discussion. In this paper, we summarized the mechanism of c-MET in NSCLC, as well as its treatment prospect and selection of testing methods.

## 1 C-MET的结构和功能

*c-MET*原癌基因存在于人类7号染色体的长臂（7q21-31）^[1]^，其蛋白产物是c-MET酪氨酸激酶受体^[2]^。c-MET蛋白的胞外区是配体识别部位，由三种类型的结构域组成：①Sema结构域，由N端500个残基折叠而成，包括整个α亚基和部分β亚基。②PSI结构域，在Sema结构域之后，由有4个保守二硫键的胱氨酸富集区域构成，横跨大约50个残基; ③IPT结构域：和免疫球蛋白样结构域相关，PSI结构域通过四个IPT结构域和跨膜螺旋相连; c-MET蛋白的胞内区具有酪氨酸激酶催化结构域，有酪氨酸激酶活性; 其蛋白受体表达于多个器官的上皮细胞，包括肝脏、胰腺、前列腺、肾脏、肌肉和骨髓，无论在胚胎期还是成人期均有表达^[3]^。c-MET的配体是肝细胞生长因子（human hepatocyte growth factor, HGF），也被称为离散因子（scatter factor, SF），由间质细胞分泌，能够促进细胞的增殖，存活，迁移，分化和形态改变^[4-9]^。

## 2 c-MET在（non-small cell lung cancer, NSCLC）中作用机制及意义

HGF/c-MET信号通路是一个复杂的、受到高度调节的过程，也是调节细胞增殖、分化和运动的重要因素，其信号通路的异常与多种肿瘤的发生、发展有关。通常通过三种机制实现：①基因层面包括基因重排、激活突变和基因扩增; ②转录上调导致的c-MET蛋白的过表达; ③通过配体依赖的自分泌或旁分泌机制使得c-MET蛋白持续激活^[4]^。在这里，我们重点探讨c-MET在NSCLC中的作用机制及意义。

在NSCLC中，当HGF和c-MET蛋白结合后，可诱导受体的同源二聚体化和位于酪氨酸激酶结构域催化环的两个酪氨酸磷残基磷酸化（Y1234和Y1235），激活了c-MET胞质内蛋白激酶结构公域中的酪氨酸激酶，从而引起c-MET羧基末端的两个酪氨酸残基磷酸化（Y1349和Y1356），进一步激活下游一系列的信号通路，主要是PI3K/Akt、MAPK、FAK、RAS和STAT。影响NSCLC细胞的增殖、存活、凋亡、侵袭、迁移和血管生成^[5]^。在NSCLC中，c-MET蛋白的异常激活可通过多种机制实现，包括：①c-MET蛋白的过表达，这可由*c-MET*基因扩增或在缺乏基因扩增的情况下转录上调所导致^[5-8]^; ②*c-MET*的基因突变，主要是N375S和T10101的序列变异^[9]^，其中，T10101的突变可能在NSCLC的恶性转化中起到重要作用^[10, 11]^; ③HGF的过表达^[12]^。

c-MET还可以和表皮生长因子受体（epidermal growth factor receptor, EGFR）相互作用影响NSCLC的进展。有研究^[13, 14]^发现，c-MET可以被EGF（EGFR的配体）激活; HGF和EGF可以协同诱导c-MET受体的磷酸化，促进下游信号转导，另外，c-MET和其他EGFR家族成员ERBB2和ERBB3（成红细胞的白血病病毒致癌基因同源染色体B2/B3）相互作用，可以引起两个受体的反式激活^[15]^，而EGFR也可以和HGF结合导致自身的二聚体化和酪氨酸激酶磷酸化，激活下游信号通路^[6]^，提示两条途径可以相互影响。此外，c-MET还和EGFR-TKIs的耐药性相关。最近的研究^[16]^发现，对EGFR-TKI获得性耐药的NSCLC往往有c-MET的扩增和过表达，提示*c-MET*的扩增和过表达是EGFR TKIs耐药的机制之一，且*EGFR*突变状态可能影响c-MET的表达水平。有人对TKIs耐药前c-MET的表达情况进行了研究，结果表明在*EGFR*突变型NSCLC中，当*c-MET*无扩增时，EGFR信号通路可以通过低氧诱导因子（hypoxia-inducible factor, HIF）-1α途径提高c-MET的表达水平^[17]^，当c-MET表达水平持续升高，超过一定界限（过表达）时，便可能引起TKIs耐药性的产生。目前的研究认为c-MET导致EGFR-TKIs耐药的机制主要有两种：①通过c-MET的扩增或过表达绕过EGFR激活ERBB3/PI3K/AKT信号通路^[18]^; ②HGF结合c-MET，而后激活GAB1信号通路^[19]^。

## 3 针对c-MET靶点治疗

目前处于临床试验阶段的c-MET的靶向药物都是针对HGF/c-MET通路的，主要包括c-MET受体激酶抑制剂（如Tivantinib、Cabozantinib、crizotinib、INC280），其机制是抑制c-MET蛋白的激活和自身磷酸化; 抗c-MET受体抗体（如onartuzumab），其机制是抑制HGF/c-MET之间的结合以及c-MET蛋白的二聚体化; 抗HGF单克隆抗体（如Ficlatuzumab、Rilotumumab、TAK701），其机制是抑制HGF/c-MET之间的结合。这些药物的研究进展已在多篇文献中有所报道，其确切疗效还有待进一步研究的证实^[6, 20, 21]^。

### 3.1 c-MET受体激酶抑制剂

#### 3.1.1 Tivantinib（ARQ197）

Tivantinib（ARQ197）具有长春新碱样作用，可阻断MET受体，使其构象失活并阻断下游信号转导。Sequist等^[22]^开展了关于Tivantinib的Ⅱ期临床试验，共入组167例之前未经过EGFR-TKI治疗的晚期NSCLC患者，被随机分为口服erlotinib（150 mg, qd）+tivantinib（360 mg, *bid*）（ET组，*n*=84）和口服erlotinib（150 mg, *qd*）+安慰剂（EP组，*n*=83）两组，主要终点为无进展生存期（progression-free survival, PFS）。研究结果显示，两组患者的中位PFS分别为3.8个月和2.3个月（HR=0.81, 95%CI: 0.57-1.16, *P*=0.24），中位OS分别为8.5个月和6.9个月（HR=0.87, 95%CI: 0.59-1.27, *P*=0.47），其主要终点PFS无统计学差异。不良反应无显著性差异，耐受性良好，最常见的为低度皮疹、腹泻，其次为乏力、恶心、呕吐、呼吸困难、贫血。探索性分析显示，在非鳞癌、*EGFR*野生型和*KRAS*突变型患者（*n*=15）中，PFS（HR=0.18, 95%CI: 0.05-0.70, *P* < 0.01），OS（HR=0.43, 95%CI: 0.12 -1.50, *P*=0.17）。PFS有明显获益。基于此，Yoshioka等^[23]^开展了关于Tivantinib在亚洲患者的Ⅲ期临床试验（ATTINTION），入组条件为经治Ⅲb期/Ⅳ期*EGFR*野生型、非鳞癌NSCLC，计划共入组460例，随机分为口服erlotinib（150 mg, *qd*）+tivantinib（360 mg, *bid*）（Tivantinib组，*n*=230）和口服erlotinib（150 mg, *qd*）+安慰剂（安慰剂组，*n*=230）两组，主要终点为总生存期（overall survival, OS）。本次研究因Tivantinib组出现肺间质性疾病（interstitial lung disease, ILD）（*n*=14，其中3例死亡）而提前终止，已入组患者（*n*=307）研究结果显示，两组中位OS分别为12.7个月和11.1个月（HR=0.891, *P*=0.427），中位PFS分别为2.9个月和2.0个月（HR=0.719, *P*=0.019），其主要终点OS没有达到统计学差异，Tivantinib组三度以上不良反应反应包括中性粒细胞减少（24.3%）、白细胞减少（18.4%）、发热性中性粒细胞减少（13.8%）和贫血（13.2%）。另外，MARQUEE研究（*n*=988）也在非鳞癌NSCLC患者中对比了erlotinib（150 mg, *qd*）+tivantinib（360 mg, *bid*）和erlotinib（150 mg, *qd*）+安慰剂的疗效，初步数据已经在2013年欧洲癌症大会上公布，结果也没有达到主要终点OS的延长。虽然Ⅱ期试验取得良好结果，但两个Ⅲ期试验却都以失败告终，其原因可能是涉及实验设计、检测方法等多方面的因素，提醒我们在以后的研究中，应更加注重对确定Tivantinib适用人群检测方法的选择，还需关注重度不良反应的发生，对已出现的不可耐受的不良反应，需探索其应对策略。

#### 3.1.2 Crizotinib

Crizotinib研发的初衷是针对c-MET酪氨酸激酶抑制剂的，其机制为通过抑制c-MET激酶与ATP的结合及结合之后的自身磷酸化而发挥作用，之后发现克唑替尼是MET/ROS/ALK的多靶点酪氨酸激酶抑制剂，且被美国食品药品管理局（Food and Drug Administration, FDA）批准用于治疗荧光原位杂交技术（fluorescence *in situ* hybridization, FISH）检测*ALK*基因重排阳性的局部晚期或转移性NSCLC，但它在c-MET阳性的晚期NSCLC中的作用依然处于探索阶段。在个案报道中，*ALK*重排阴性但*c-MET*扩增阳性（FISH检测）的患者服用克唑替尼后展现出了良好的临床效应^[24]^; 2014年美国临床肿瘤学会年会（American Society of Clinical Oncology, ASCO）会议报道了克唑替尼在晚期*c-MET*扩增的NSCLC中的疗效和安全性，该研究使用FISH检测*c-MET*扩增状态，入组患者按MET/GEP7（7号染色体着丝粒）比率分为三组：1.8-2.2（低，*n*=2），2.2-5（中，*n*=6）和 > 5（高，*n*=6）。这项研究是一项正在进行的克唑替尼的I期研究中的一部分（NCT00585195）。患者服用克唑替尼250 mg bid，采用实体瘤疗效评价标准（Response Evaluation Criteria in Solid Tumors, RECIST）V1.0评估缓解率。结果显示，低、中、高度扩增患者的有效率分别为0、17%和67%。常见的不良事件包括腹泻（50%）、恶心（31%）、呕吐（31%）、周围性水肿（25%）和视觉障碍（25%），大多数不良事件为1度。研究者认为克唑替尼在*c-MET*基因扩增型NSCLC患者中呈现出了良好的应用前景，克唑替尼250 mg bid患者普遍能够耐受，不良事件亦在可接受范围。该结果支持对*c-MET*扩增NSCLC进行克唑替尼治疗的深入研究; 2015年ASCO会议上展示了克唑替尼治疗*de novo* c-Met过表达的晚期NSCLC试验。研究者使用免疫组化技术（immunohistochemistry, IHC）检测晚期NSCLC患者*de novo* c-Met表达情况，FISH技术检测基因拷贝数变化，同时还检测了EGFR、ALK、KRAS、ROS1（均为阴性）。截止数据分析，共有24例c-Met IHC过表达患者接受克唑替尼治疗，其中19例可供评估缓解情况。11例患者部分缓解（partial response, PR），3例病情稳定和5例进展。有1例患者出现三级副反应QT间期延长。还有1例患者死于间质性肺病，但未排除克唑替尼的影响。其他常见的副反应为1级-2级副反应，如恶心（14/19）、厌食（14/19）、呕吐（10/19）和视力障碍（6/19）。目前结果提示，克唑替尼也许是*de novo* c-Met过表达的NSCLC患者较好的治疗方法，试验样本量仍在扩大，我们期待以后的结果。另外，该研究中缓解的患者c-Met IHC均为高表达，其中有8例FISH检测为阳性，提示用IHC检测*c-Met*的有效性可能优于FISH，我们需要确定出最敏感的检测*c-MET*扩增方法，以便更好地选出适合接受克唑替尼治疗的*c-MET*扩增晚期NSCLC患者。

### 3.2 抗c-MET受体抗体

Onartuzumab（OA-5D5, OAM4558g, Met MAb）是一种人源性抗MET单克隆抗体。主要通过抑制HGF/c-MET之间的结合及c-MET二聚化发挥抗肿瘤作用。在Spigel等^[25]^开展的一项关于onartuzumab的Ⅱ期临床试验中，共纳入137例经治的晚期NSCLC患者，随机分为erlotinib（150 mg/kg, *qd*）+ onartuzumab（15mg/kg，静注）（*n*=69）和erlotinib（150 mg/kg, *qd*）+安慰剂（*n*=68）两组，主要终点为PFS，研究结果显示受试者人群中两组PFS和OS均无改善（n=137; PFS: HR=1.09; *P*=0.69; OS: HR=0.80; *P*=0.34）; 但在MET阳性患者（*n*=66）中erlotinib+onartuzumab组PFS (中位PFS: 1.5个月*vs* 2.9个月; HR=0.53;*P*=0.04）和OS（中位OS: 3.8个月*vs* 12.6个月; HR=0.37;*P*=0.002）均有显著延长，基于此，Spigel等^[26]^开展了关于onartuzumab的随机多中心安慰剂对照的Ⅲ期临床试验（MET-lung），入组患者为MET IHC阳性的晚期NSCLC经治患者，共入组490例患者，按1:1被随机分为两组，一组患者静注onartuzumab（15 mg/kg）+口服erlotinib（150 mg, *qd*）; 另一组静注安慰剂+口服erlotinib（150 mg, *qd*），每21天一个疗程，研究主要终点为OS。入选患者还会根据MET IHC临床评分（2+ *vs* 3+），经治次数（1 *vs* 2），组织学类型（非鳞癌*vs*鳞癌）和EGFR活化状态（突变型*vs*野生型）进行分层。研究结果显示，onartuzumab组和安慰剂组中位OS分别为6.8个月和9.1个月（HR=1.27, 95%CI: 0.98-1.65, *P*=0.07），中位PFS分别为2.6个月和2.7个月（HR=0.99, 95%CI: 0.81-1.2, *P*=0.92），且OS和PFS在各亚组分析中也无阳性结果; 最常见的不良反应为腹泻（39%）和皮疹（39%），外周水肿（22% *vs* 8%）和低蛋白血症（17% *vs* 4%）更多见于onartuzumab组。虽然本次研究没有得到阳性结果，但MET受体抑制剂依然具有潜在的临床应用价值，根据*MET*基因突变、基因扩增和过表达的特点以及他们之间的关系，从而找出合适治疗人群正确的生物标志物至关重要。

### 3.3 抗HGF单克隆抗体

其抗肿瘤机制是通过与HGF配体结合，阻碍HGF和c-MET的结合，从而阻断下游一系列的信号转导。目前还没有关于此类药物大样本临床试验的研究，对于其临床疗效的判定还需进一步探索，根据其他药物临床试验的结果，提醒我们在后续研究过程中，需注意合适治疗人群的选择标准，以及对可能出现的重度不良反应的应对策略。

## 4 c-MET的检测

由于在NSCLC患者中可存在*c-MET*基因的突变、扩增和过表达; 故其相应的检测方法有直接测序法、实时荧光定量PCR（real-time fluores cence quantitative PCR, RTFQ-PCR）、FISH和IHC，通过分析*c-Met*基因和c-MET蛋白筛选出适合c-MET抑制剂应用的获益人群十分必要。

### 4.1 基因突变

2015年ASCO大会上报道了关于*c-MET*基因突变的研究（#11007），指出它常出现在肺腺癌中，发生率为3%，其最常见的变异类型是外显子14的供体位点剪接突变（45.1%），且病历报道/系列研究显示具有此变异的NSCLC患者对MET抑制剂治疗有反应。直接测序法检测*c-MET*突变具有高度的准确性，且不必明确*c-MET*基因突变位点所在的具体位置，可以检测出其他潜在的外显子突变位点，2015年2月美国Dana-Farber癌症研究所报告了全球首例*MET*基因剪接点突变的晚期肺腺癌对克唑替尼有效。该患者EGFR、*KRAS*、*ALK*、*ROS1*和*RET*等驱动癌基因均为阴性，但靶向二代测序（next generation sequencing, NGS）显示*MET*基因14号外显子剪接点突变（c.2887-18_2887-7del12），IHC提示50%癌细胞表现为磷酸化c-Met蛋白2+的阳性反应，与c-Met蛋白的活化状态相一致。这一结果提示Crizotinib对*c-MET*基因14号外显子突变的NSCLC患者有效，但这只是个案报道，其临床应用价值还需大样本试验的证实。另外，有研究^[27]^指出，在NSCLC中*c-MET*突变的发生率较低，扩增和过表达更常见，而直接测序法无法检测出*c-MET*拷贝数目的变化，提示其不能筛选出所有适合c-MET靶向治疗的获益人群; 所以如果想通过检测*c-MET*基因突变的状态来筛选合适的治疗人群，就需要明确基因突变和基因扩增之间的关联性，以及他们所引起的蛋白过表达的量对肿瘤生长的影响程度，这也为我们以后的研究提供了方向。

### 4.2 基因扩增

RTFQ-PCR和FISH均可用于c-MET扩增的检测，前者检测速度更快，但是对基因组DNA片段的质量要求较高，而现有的提取方法得到的基因组DNA片段质量较差^[28]^，因此二者相比，FISH的应用更广泛。FISH法可通过检测MET位点和CEP7（作为对照）来判定*MET*基因拷贝数（*MET* gene copies number, MET GCN），其评估标准可依据两个方面：①MET/CEP7的比值; ②每一个细胞的基因拷贝数量及其阳性细胞所占总细胞比例。2014年ASCO会议报道了克唑替尼在晚期*c-MET*扩增的NSCLC中的疗效和安全性（前已述及），该研究使用FISH检测*c-MET*扩增状态，该研究结果肯定了FISH法在筛选适合MET抑制剂治疗人群时的作用，但以此标准判定的MET FISH阳性率较低; 在Sequist等^[22]^设计的一项关于Tivantinib的Ⅱ期临床试验中（前文已述及），评估MET FISH阳性的标准为≥40%的细胞有≥4个基因拷贝数。研究结果显示，在所有进行检测的患者（*n*=142）中只有3例患者MET/CEP7大于2，而有37例患者（26%）的MET GCN≥4个; 亚组分析结果提示，随着MET GCN的提高，ET组获益程度也相应提高，MET GCN≥2、3、4、5的PFS HRs分别为0.92、0.75、0.71、0.42，同时OS也有此趋势，但两者均无统计学差异。这两项研究结果提示，FISH法检测*c-MET*扩增状态的评估标准的准确性可能更倾向于MET/CEP7的比值，但还需要大样本临床试验的证实，另外，对于MET/CEP7最佳比值和MET抑制剂临床获益的相关性也需深入研究。

### 4.3 蛋白过表达

IHC可用于检测c-MET蛋白的过表达情况。MET IHC以抗-c-MET（SP44）兔单克隆抗体（#7904430, Ventana Medical Systems, Tucson, AZ）为一抗，以抗-RbOmniMap DAB（#760149, Ventana Medical Systems）为二抗，阳性标准以两种不同的方法判定：①H-评分，这一评分根据样本的染色强度（0-4）和阳性肿瘤细胞所占细胞总数的比例（0-100%）而判定，其分数为0-400不等，只要H-评分大于200便可判定为阳性; ②将肿瘤细胞分为MET高表达和低表达两类，其具体判定标准为：3+（≥50%的肿瘤细胞呈强阳性）; 2+（≥50%的肿瘤细胞呈阳性/弱阳性且 < 50%的肿瘤细胞呈强阳性）; 1+（≥50%的肿瘤细胞呈弱阳性且阳性细胞数 < 50%）; 0（无染色或任何强度染色的肿瘤细胞数均 < 50%）; 2+或3+被定义为MET高表达，0或1+倍定义为MET低表达（Metmab标准）^[25, 29]^。2015年ASCO会议上展示了克唑替尼治疗*de novo* c-Met过表达的晚期NSCLC试验（前已述及），MET IHC的判定依据Metmab标准; 该研究中缓解的患者c-Met IHC均为高表达，其中有8例FISH检测为阳性，提示用IHC检测c-Met的有效性可能较FISH法能更敏感地筛选出合适Crizotinib的治疗人群; 另外，在一项关于onartuzumab的Ⅱ期临床试验中（前已述及）^[25]^，137例经治晚期NSCLC患者，被随机分为onartuzumab+erlotinib（*n*=69）和erlotinib+安慰剂（*n*=68）两组，通过MET IHC的方法检测MET蛋白表达量，判定方法为Metmab标准，该研究结果显示，onartuzumab组在MET IHC阳性的患者中有PFS和OS的显著延长; 但MetMab的Ⅲ期临床试验（METLung）却以失败告终，且各亚组也无阳性结果，提示MET IHC不能特异地筛选出合适的靶向治疗人群，需要更精准的检测方法定义c-MET的阳性表达。最近一项研究^[30]^发现，无论MET蛋白表达还是*c-MET*基因扩增，均和MET蛋白磷酸化没有显著相关性，故可能两者都不是选择c-MET靶向治疗人群的最佳方法，他们认为，由于在临床上用IHC的方法检测的是c-MET总蛋白的表达量，而MET蛋白只有在被异常激活以后才会通过激活下游通路在NSCLC的发生发展中起作用，故通过IHC的方法分析MET蛋白的磷酸化状态比单纯检测MET蛋白表达量更适合选择c-MET靶向治疗的人群。所以在应用IHC方法时，需注意MET蛋白表达量界限的判定标准，并确定是否需要检测其磷酸化的状态; 另外，H-评分和Metmab标准在MET IHC中应用前景的比较也许需研究探讨。

通过上述对基因突变、基因扩增和蛋白过表达三种相应检测方法的描述，似乎可以看出检测基因突变和基因扩增的特异性较蛋白表达更好，而IHC法灵敏度更高，对于最佳方法及其判定标准的选择，还需我们明确基因突变、扩增以及蛋白过表达之间的具体联系及其和肿瘤细胞生长的关系。此外，在对c-MET进行检测时，还要注意以下几点：①福尔马林固定的石蜡包埋肿瘤组织是检测致癌基因分子改变很好的标本，但其所提取的基因组DNA片段的质量较低，故提取过程有待优化^[28]^。②在用微创的方式获取组织标本时，要注意获取足够的标本进行检测，不要混进正常或坏死的组织^[31]^。③当无法通过手术等方式获取活检标本时，外周血循环肿瘤细胞（circulating tumor cell, CTC）或外周血游离肿瘤DNA（cfDNA）或可作为其替代品，但还需要进一步的研究证实^[32, 33]^。④随着疾病进展或对治疗的反应，肿瘤的基因组成可能会发生改变，所以疾病进展后再次取活检（组织学或细胞学）可能会为下一步治疗提供更准确的检测结果^[27]^。

## 5 小结

HGF/c-MET信号通路的异常调节在NSCLC的发生发展以及EGFR-TKI耐药方面发挥着重要作用，针对c-MET治疗的研究也越来越多，并展现出了一定的应用前景，但还有很多问题亟待解决。目前研发的c-MET抑制剂有很多种，虽然从分子机制的角度分析，这些药物均应表现出良好的效果，但在临床试验的过程中还是呈现出了很多问题，比如c-MET抑制剂应用时机、应用剂量的选择以及安全性评估等; 另外，在NSCLC中，关于c-MET与EGFR、c-MET与ALK表达之间的联系以及相应治疗策略选择的研究已有很多，但具体治疗措施尚未确定，还有待进一步探索。综合多项临床试验来看，影响c-MET抑制剂治疗效果好坏的关键因素之一在于对c-MET检测方法的选择，是否能确定出有效筛选c-MET扩增晚期NSCLC患者治疗获益人群的检测方法及判定标准至关重要，仍需进一步探索。
